# Onco-fertility and personalized testing for potential for loss of ovarian reserve in patients undergoing chemotherapy: proposed next steps for development of genetic testing to predict changes in ovarian reserve

**DOI:** 10.1186/s40738-021-00105-7

**Published:** 2021-06-30

**Authors:** Bei Sun, John Yeh

**Affiliations:** 1 Sackler School of Medicine, New York State/American Program of Tel Aviv University, Tel Aviv University, Ramat Aviv 69978, Tel Aviv, Israel; 2 Division of Reproductive Endocrinology and Infertility, Department of Obstetrics & Gynecology, University of Massachusetts Medical School, UMass Memorial Medical Center, 119 Belmont Street, Worcester, MA 01605 USA

**Keywords:** Oncofertility, Fertility preservation counseling, Ovarian reserve testing, Chemotherapy, Ovarian damage

## Abstract

Women of reproductive age undergoing chemotherapy face the risk of irreversible ovarian insufficiency. Current methods of ovarian reserve testing do not accurately predict future reproductive potential for patients undergoing chemotherapy. Genetic markers that more accurately predict the reproductive potential of each patient undergoing chemotherapy would be critical tools that would be useful for evidence-based fertility preservation counselling. To assess the possible approaches to take to develop personalized genetic testing for these patients, we review current literature regarding mechanisms of ovarian damage due to chemotherapy and genetic variants associated with both the damage mechanisms and primary ovarian insufficiency. The medical literature point to a number of genetic variants associated with mechanisms of ovarian damage and primary ovarian insufficiency. Those variants that appear at a higher frequency, with known pathways, may be considered as potential genetic markers for predictive ovarian reserve testing. We propose developing personalized testing of the potential for loss of ovarian function for patients with cancer, prior to chemotherapy treatment. There are advantages of using genetic markers complementary to the current ovarian reserve markers of AMH, antral follicle count and day 3 FSH as predictors of preservation of fertility after chemotherapy. Genetic markers will help identify upstream pathways leading to high risk of ovarian failure not detected by present clinical markers. Their predictive value is mechanism-based and will encourage research towards understanding the multiple pathways contributing to ovarian failure after chemotherapy.

## Search strategy and selection criteria

A total of 373 papers were reviewed. For the section on mechanism of chemotherapy-induced damage, the authors performed PubMed searches using the following key words: chemotherapy, induced, ovarian insufficiency, mechanism. No time limits were placed on the time of publication and 87 results were obtained. Animal and clinical studies as well as references from review articles that evaluate the mechanism of chemotherapy-induced ovarian damage were selected. For the sections on genetic association with ovarian insufficiency, the authors performed PubMed searches using the following key words: genetic, variants, ovarian insufficiency. No time limits were placed on the time of publication and 286 results were obtained. Animal and clinical studies that report a genetic association with ovarian insufficiency were selected.

## Introduction

The probability of premenopausal women to develop any type of invasive cancer is approximately 6%, among which breast cancer is the most common [1]. Chemotherapy as part of the treatment induces ovarian dysfunction [2]. In 2013 globally, the number of women of reproductive age was 1.8 billion and this number is expected to grow to 2 billion by 2025 [3]. Based on these statistics, we extrapolate that more than 100 million women worldwide are at risk to chemotherapy-induced ovarian dysfunction and may seek fertility preservation. A mixed retrospective and prospective study of 102 women who underwent chemotherapy reported a 77.9% incidence of irreversible amenorrhea 12 months after completion of chemotherapy [4]. Fertility preservation counseling remains challenging and centers around the ovarian reserve of each patient [5]. Various chemotherapy regimens each presents with a different level of risk of loss of ovarian reserve [6]. In addition, patients clinically present with variable susceptibility to ovarian dysfunction. Helping patients understand their baseline and predicted post-chemotherapy reproductive potential requires clinical markers that accurately assess individual susceptibility to ovarian dysfunction.

Current ovarian reserve testing relies mainly on biochemical tests and ultrasound imaging [7]. For example, molecular markers such as anti-mullerian hormone (AMH) and inhibin have been reported to be associated with chemotherapy-induced ovarian damage and reproductive aging [8–11]. While such tests have a high predictive value in high-risk populations, they are not routinely used in clinics to serve the general population. An editorial has suggested the use of genetic markers to predict outcome of ovarian function after chemotherapy [12]. As the genetic basis of premature ovarian insufficiency continues to be explored [13–15], genetic markers can be used in addition to ovarian reserve testing to help establish a more comprehensive baseline. Genetic and molecular pathways involved in chemotherapy-induced ovarian damage have been discussed in several studies [2, 16, 17]. We review the mechanism of ovarian damage and the associated genes as potential markers. We also review studies that investigate frequency of genetic mutations associated with premature ovarian dysfunction. An upstream regulator gene involved in chemotherapy-induced damage pathways whose mutations are frequently found would make an ideal candidate as a genetic marker. It can potentially help clinicians separate patients into the following categories for appropriate counseling: 1) higher theoretical genetic risk of loss of ovarian reserve at baseline 2) Increased theoretical ovarian reserve loss risk due to use of chemotherapy because of genetic mutations 3) lower theoretical genetic risk.

## Chemotherapy and genetic predisposition to ovarian insufficiency

### Mechanism of chemotherapy-induced damage

**Fig. 1:**
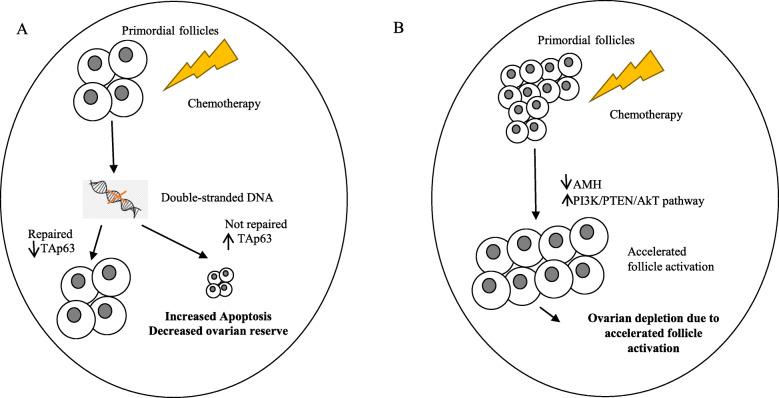
Two mechanisms of chemotherapy-induced ovarian reserve depletion: increased apoptosis and accelerated follicle activation. **A.** DNA lesions induced by chemotherapy activates repair pathways. Failure to repair the lesions induces TAp63-mediated apoptosis of germ cells. **B.** Chemotherapy destroys growing follicles leading to a reduction in AMH level. This results in upregulation of PI3K/PTEN/AkT pathway which over-recruits primordial follicles. The accelerated activation of follicles ultimately leads to depletion of ovarian reserve [2]

Ovarian dysfunction induced by chemotherapy was first reported in 1970s [18, 19]. The mechanism of damage of these agents and protective measures have since been investigated [20–24]. Direct toxicity leading to apoptosis of oocytes and granulosa cells as well as accelerated follicle activation have emerged as mechanisms of chemotherapy-induced ovarian dysfunction (Fig. 1). In the case of breast cancer for example, more chemotherapeutic agents and more combinations of these agents have been approved over time. The effect of these agents on fertility and the mechanism of their damage on the ovaries have been investigated (Table 1). Both apoptosis and follicle overactivation seem to play a role in ovarian reserve depletion in the use of some agents. To what percentage of ovarian damage is each mechanism responsible is not understood. It is likely to be specific to each chemotherapeutic agent and should be studied quantitatively in animals. Alternative additional mechanisms of ovarian damage should also be explored.

**Table 1:** Chemotherapeutic agents used in breast cancer treatment cause ovarian damage

Chemotherapeutic Agent	Biological Target	Mechanism of Ovarian Damage	Reference
Taxanes	microtubule	Apoptosis and suppression of follicle development	[25, 26]
Anthracyclines	DNA	Apoptosis, atresia and overactivation of ovarian follicles	[27, 28]
Platinum agents	DNA	Both apoptosis and overactivation of ovarian follicles	[29]
Vinorelbine	microtubule	Unknown or none	[30]
Capecitabine	DNA	Unknown or none	[31]
Gemcitabine	DNA	Apoptosis of pre-antral/antral follicles	[32]
Ixabepilone	microtubule	Unknown	N/A
Eribulin	microtubule	Unknown	N/A

#### Apoptosis of oocytes and granulosa cells

Chemotherapeutic agents may induce DNA alterations. These agents have been reported to disproportionally affect follicles in various stages and stromal cells [16, 32]. Double stranded-DNA breaks are among the most severe DNA lesions induced by these agents [33]. These lesions effectively activate DNA repair system leading to either cell survival or apoptosis (Fig. 1A). Studies support apoptosis as an important mechanism underlying chemotherapy-induced depletion of ovarian reserve through knockout (KO) experiments of pro-apoptotic factors in animals. For example, Ngyuen et al. experimented with mouse knockout models of p53 upregulated modulator of apoptosis (PUMA) and its transcriptional activator TAp63 [34]. They exposed wildtype (WT), PUMA KO, and TAp63 KO mice to either cyclophosphamide or cisplatin treatments. Complete destruction of primordial follicles was observed in WT mice in contrast to close to 100% preservation of primordial follicles in PUMA KO mice. Preservation of primordial follicles in TAp63 KO mice was observed in cisplatin-treated mice. Another study demonstrated mechanism of germinal vesicle oocyte apoptosis induced by doxorubicin through activation of caspase-12 and inactivation of DNA repair machinery such as poly-ADP ribose polymerase (PARP) [27]. Regulation of apoptosis of granulosa cells have also been studied extensively in animal models [35–39].

#### Accelerated follicle activation

Accelerated primordial follicle expenditure emerged as another important mechanism of chemotherapy-induced depletion of ovarian reserve (Fig. 1B). A study on cyclophosphamide-treated mice revealed that a wave of induced follicle activation rather than apoptosis is responsible for the rapid loss of ovarian reserve [40]. Maintenance of dormancy in primordial follicles underlies the reproductive lifespan of women. It is controlled by multiple signaling pathways that have been uncovered through animal studies [41–43]. Phosphatidylinositol 3-kinase (PI3K)/Akt signaling pathway negatively regulated by phosphatase and tensin homolog (PTEN) has been studied in mice and shown to be important in control of dormancy and implicated in chemotherapy-induced follicle burnout [43–45]. Suppressors of this follicle-activating pathway such as forkhead box O3a (FOXO3a), tuberous sclerosis protein 1 (TSC1) and tuberous sclerosis protein 2 (TSC2), when deactivated through phosphorylation, accelerates follicle recruitment and growth [41]. Phosphorylation of molecules in the pathway such as Akt and mTOR has a similar accelerating effect [43, 46, 47]. Chemotherapeutic agents have been shown to upregulate these activating pathways. For instance, cisplatin has been shown to decrease PTEN levels, leading to increased phosphorylation of Akt and subsequent pan activation of dormant primordial follicles in mice [48]. Another study showed that mTOR inhibitor cotreatment with cyclophosphamide preserves follicle count and fertility in mice through down-regulation of PI3K/Akt/mTOR pathway [49]. There has been a lack of human studies to provide support for this mechanism. So far, an in vitro study of human ovarian follicle exposed to metabolites of cyclophosphamide showed enhanced follicle activation [50]. In addition, a recent cohort study of 96 women showed a significant reduction in nuclear expression of FOXO3a in primordial follicle oocytes in the ovaries of women exposed to alkylating agent chemotherapy, supporting accelerated follicle activation as a major mechanism of ovarian reserve depletion after exposure to alkylating agents [51].

### Genetic susceptibility to chemotherapy-induced damage

#### Genes in DNA damage repair

Homologous recombination and non-homologous end joining are mechanisms involved in repair of chemotherapy-induced severe DNA lesions in primordial follicles. Genetic defects that compromise the two repair pathways potentially increase patients’ susceptibility to ovarian failure after chemotherapy. Major genes implicated in this susceptibility are discussed in the order of number of clinical evidences. BRCA1 and BRCA2 genes are crucial in repairing double-stranded DNA breaks. Their mutation carriers have a high risk of developing cancer. Furthermore, they often have fertility-related problems [52]. A cross-sectional study of 693 women showed that BRCA1 mutation carriers had on average 25% lower AMH concentrations than non-carriers, suggesting that BRCA1 carriers had lower ovarian reserves than non-carriers [53]. No evidence of association was found between AMH concentration and BRCA2 mutation status in the same study. Another study surveyed 908 matched pairs of BRCA1 mutation carriers and non-carriers and found that carriers experienced earlier menopause [54]. Consistent with these findings, Oktay et al. reported a lower ovarian response rate and a lower number of eggs produced in BRCA1 mutation carriers compared to non-carriers under ovarian stimulation [55]. No association between BRCA2 mutations and the probability of low ovarian response was found in the study. Current studies consistently point to a strong association between BRCA1 mutation status and propensity of ovarian failure. For BRCA2, certain mutations are associated with total failure of ovarian development and appears to result in a more widespread ovarian damage either in utero or early stage of development [56, 57]. Minichromosome maintenance complex component 8 and 9 (MCM8, 9) are another two genes involved in DNA repair that is associated with primary ovarian insufficiency (POI) [58]. Stromal antigen 3 (STAG3), a meiosis-specific gene expressed only in human testis and ovary, is important in DNA repair [59]. A recent study found two novel in-frame variants of STAG3 that are associated with primary ovarian insufficiency in two sisters from a five-generation consanguineous Han Chinese family [60]. Similar to STAG3 in which clinical evidence associating its mutation to ovarian dysfunction is limited to case studies, helicase for meiosis 1 (HFM1), nucleoporin 107 (NUP107) and synaptonemal complex central element protein 1 (SYCE1) were identified as candidate genes in DNA repair that are implicated in ovarian insufficiency [13, 61–63].

#### Genes in apoptosis

The process of apoptosis is highly regulated and plays a crucial role in maintaining a pool of primordial germ cells. During embryonic phase, germ cells that incurred replication errors are eliminated [64]. Furthermore, during each menstrual cycle, there is atresia of follicles other than the dominant follicle. Dysregulation of the process not only leads to a diminished ovarian reserve but also an increased susceptibility to ovarian failure upon exposure to chemotherapy. A study showed that an anti-apoptotic gene bcl-2 knockout transgenic mice have markedly reduced number of primordial germ cells compared to that of control [65]. Other candidate genes involved in the process start to emerge thanks to next generation sequencing [13]. Nanos C2HC-Type Zinc Finger 3 (NANOS3) encodes for an RNA-binding protein that represses apoptosis important in maintaining a healthy pool of primordial germ cells. A missense variant of NANOS3 was identified in a study of Chinese women with primary ovarian insufficiency, and the level of NANOS3 protein was shown to correlate with the number of primordial germ cells [66]. A homozygous mutation of NANOS3 was identified in a different study in two sisters with primary amenorrhea [67]. Another candidate gene, progesterone receptor membrane complex 1 (PGRMC1) which suppresses apoptosis through the action of progesterone, was found to be associated with POI [13, 14]. A study that screened 67 women with idiopathic primary ovarian failure identified a missense mutation of progesterone receptor membrane component 1 (PGRMC1) [68].

#### Genes in follicular activation and development

Animal and clinical studies have shown that genetic mutations involved in follicular activation process are associated with primary ovarian failure and likely an increased susceptibility to ovarian failure after chemotherapy [13–15]. FOXO3a is an important suppressor of follicle activation. A study showed that FOXO3a knockout mice had early depletion of ovarian follicles compared to control mice [46]. Screening of 90 women with primary ovarian insufficiency was done by Watkins et al. and rare, potentially causal variants of FOXO3a and FOXO1a were identified [69]. A few years later, another study analyzed FOXO3 mutations in 114 Chinese women with premature ovarian failure and identified six new variants that might cause early follicle depletion [70]. Genetic mutations involved in early follicle development are also implicated in an increased susceptibility to ovarian failure after chemotherapy. Bone morphogenic protein 15 (BMP15) is expressed exclusively in oocytes and was shown to be an important regulator of ovulation rate and ovarian reserve [71]. Small-scale clinical studies in India, Italy and Syria consistently identified genetic variants in women with premature ovarian failure [72–74].

## Protective genetic variants against chemotherapy induced ovarian insufficiency

Potential ovarian protective effects of genetic variants have been reported. A few studies discussed this aspect of genetic associations with POI. For example, a study found a significantly reduced allele frequency of inhibin alpha gene promoter in a group of patients from New Zealand and Slovenia with POI compared to the control group and suggested a potential protective effect of the allele against POI [75]. A similar study with a larger scale conducted in Italy and Germany suggested a similar protective effect of the rare allele [76]. Another group of studies investigated the protective effect of resveratrol on POI. One such study reported an increased level of MVH,OCT4, SOD2, GPx, and CAT detected after the treatment with resveratrol both in vivo and in vitro [77]*.* Such protective effect was found to be dose-dependent [78]. This suggests that genetic pathways regulating germline stem cell proliferation and antioxidant enzymes may play a role in protection against development of POI. In addition, a Korean research group reported certain haplotype of microRNA occurred less frequently in patients with POI compared to that in control subjects and further suggested a potential protective effect of these haplotypes [79]. While loss of function of pro-apoptotic genes has been reported to prevent follicle depletion during chemotherapy, such genetic variants may not preserve germline genome integrity [34, 80].

## Frequency of genetic variants in primary ovarian insufficiency

Clinical studies have shown a group of gene variants that appear at a relatively high to medium frequency in patients with POI (Table 2). Many of them are X chromosomal defects and have been shown to be specific to certain ethnic groups. For example, a study conducted in the UK enrolled over two thousand women who experienced menopause before age of 46 to investigate the frequency of premutation in Fragile X Mental Retardation 1 (FMR1) gene characterized by 55–200 CGG repeats [98]. FMR1 premutation was shown to appear at around 2% frequency in patients presented with POI compared to 0.4% in the control group. The FMR1 gene is essential for various structures associated with the female reproductive system. It impacts the establishment and the maintenance of cells such as granulosa cells, oocytes, and luteal cells. The gene also plays an indirect role in estrogen secretion by its impact on follicle-stimulating hormone (FSH) levels in the menstrual cycle. Yang et al. found that FMR1 gene expression plays a role in germline stem cells, using Drosophila as a model [99]. Their results showed that the Drosophila FMR1 protein is associated with maintaining oocyte germline stem cells and suppressing differentiation [99]. In humans, the FMR1 premutation is linked to the abnormal levels of FSH secreted from the hypothalamic-pituitary-ovarian axis [100]. Welt and colleagues measured the hormone levels across the menstrual cycle of human females with the FMR 1 premutation. They found that the menstrual cycle was shorter for the FMR1 carriers, especially the follicular phase. Increased levels of FSH was identified during the entire menstrual cycle, and decreased levels of inhibin B, inhibin A, and progesterone were found [100]. It was hypothesized that the decreased levels of inhibin cause a decrease in the negative feedback system, hence an increase in the FSH secretion from the pituitary gland. The imbalance of hormones may cause abnormality in follicular development. The anomalous menstrual cycle associated with FRM1 premutation is therefore a cause of dysregulation of oocyte development.

**Table 2:** Genetic Variants Associated with POI

Mechanism	Gene	Subject Demographics	Subject Sample Size & Selection Criteria	Variant Frequency	Reference
Follicle Development	NOBOX	Caucasian, Senegalese, Bantu [81] Caucasian, African [82]	178 women diagnosed with idiopathic POI and 362 ethnic-matched women control [81] 213 women diagnosed with idiopathic POI and 362 ethnic-matched women control [82]	6.2% in POI group 0% in control group [81] 7% in POI group 0% in control group [82]	[81, 82]
	FIGLA	Chinese	100 women diagnosed with POI and 304 healthy women between 30 and 62 years old with regular menses and no history of infertility as control	4% in POI group 0.3% in control group	[83]
	BNC1	Chinese	82 women diagnosed with POI and 332 healthy female control	4% in POI group 0% in control group	[84]
	SOHLH1	China, Serbia	364 Chinese women and 197 Serbian women diagnosed with POI 400 Chinese and 200 Serbian women with regular menses and normal FSH level as control	2.2% in Chinese POI group 0.9% in Chinese control group 0% in Serbian POI group 0% in Serbian control group	[85]
	SOHLH2	China, Serbia	364 Chinese women and 197 Serbian women diagnosed with POI; 222 Chinese and 200 Serbian women with normal menses and normal FSH level as control	2.2% Chinese POI group 0% in Chinese control group 2% in Serbian POI group 0% in Serbian control group	[86]
	FOXO3A/FOXO1A	China, New Zealand, Slovenia	114 Chinese patients diagnosed with POI and 100 control subjects under the age of 40 with proven fertility, normal menstrual cycle and ovarian morphology [87] 30 patients from New Zealand and 60 patients from Slovenia diagnosed with POI and 60 healthy control subjects [69]	13% in POI group (FOXO3) 0% in control group (FOXO3) [87] 2.2% in POI group (FOXO3A) 0% in control group (FOXO3A) 1.1% in POI group (FOXO1A) 0% in control group (FOXO1A) [69]	[69, 87]
Follicle Development	BMP15	US (Caucasian)	166 Caucasian women diagnosed with POI and 211 controls (95 women with menopause beyond 50 years of age, 86 women and 30 men from the general population)	2.1% in POI group 0% in control	[88]
	KHDRBS1 (or Sam68)	Chinese	215 women diagnosed with POI and 400 women over age of 40 not diagnosed with POI with a history of regular menstrual cycle	0.04% in POI group 0% in control group	[89]
FMR1 Premutation (55–200 CGG repeats)	Unknown	UK	254 women presented with POI and 1915 controls selected either as postmenopausal at entry with a menopausal age of 46 years or older (74.3%) or premenopausal and entered the study at 46 years or older (25.7%)	2% in POI group 0.4% in control group	[90]
DNA Damage Repair	BRCA1	UK	2028 women diagnosed with breast cancer before age of 55 between 1991 and 1996 prevalence calculated based on a mathematical model	1.2% in cancer group; 0.09% in general population	[91]
	MCM8/9	US	155 women diagnosed with POI Control group data from public database such as exome variant server	2% in POI group (MCM8) 0% in control group (MCM8) 5% in POI group (MCM9) 0% in control group (MCM9)	[92]
	FANCM	Chinese	200 patients diagnosed with POI and 200 age-matched women with regular menses an normal FSH level as control	0.4% in POI group 0% in control group	[93]
Apoptosis Regulation	NANOS3	Chinese, Caucasian, Brazilian	80 Chinese women and 88 Caucasian women diagnosed with POI and 63 healthy Chinese and 63 healthy Caucasian control subjects [94] 30 Brazilian women diagnosed with POI and 185 women with normal fertility as control [95]	0.14% in Chinese POI group 0.09% in Chinese control group 0.09% in Caucasian POI group 0.03% in Caucasian control group [94] 0% in POI group 0% in control group [95]	[94, 95]
	PGRMC1	China	196 nulligravida women diagnosed with POI without family history of POI or X chromosome abnormalities and 200 healthy women with regular menstrual cycle and no known history of infertility before age of 40 years	0.51% in POI group 0% in control group	[96]
HFM1	Meiosis	Chinese	69 women diagnosed with POI and 316 controls matched for ethnic background, sex and age	2.9% in POI group 0% in control group	[97]

*POI Primary ovarian insufficiency characterized by onset of menopause before age of 40 with elevated FSH level

The frequency of BRCA1 mutation highly associated with POI, was also investigated in the UK in a study with over two thousand women diagnosed with breast cancer between 1991 and 1996 [91]. The study found a frequency of 1.2% of BRCA1 mutation in the patient group compared to 0.4% in the control group. Other studies conducted so far are small-scale that enrolled patients in the low hundreds. A few genetic variants such as MCM8/9, BMP15, FOXO3 and SOHLH1 have been found to occur around 2% frequency in POI patients compared to close to 0% in control groups (Table 2). Genetic variants such as NANOS3, FOXO1A and PGMRC1 have been found to appear at frequency lower than 1.1% in various ethnic groups. Autosomal defects underlying a group of complex diseases ranging from metabolism to autoimmune disorders, in rare occasions, are associated with POI.

## Clinical considerations

### Candidate genetic markers

There are several advantages of using genetic markers complementary to the current ovarian reserve markers of AMH, antral follicle count and day 3 FSH as predictors of preservation of fertility after chemotherapy. Genetic markers will help identify disturbances in upstream pathways leading to high risk of ovarian failure that may be missed by molecular markers. An ideal genetic marker should have a high-frequency variant specific to patients who experience ovarian failure after chemotherapy. In addition, the basic science of the associated genetic pathways should be investigated and understood. Genes upstream in these pathways could be selected for additional insights. Based on our literature review, such candidate genes associated with POI and chemotherapy-induced ovarian damage mechanisms have been identified (Table 2). The difference between the prevalence of the gene variants in the POI patients and in the healthy patients affect the positive and negative predictive values of the genetic markers. FMR1 and BRCA 1 testing are performed routinely in genetic clinics. Large-scale studies of FMR1 premutations and BRCA1 mutations revealed their prevalence to be 2 and 1.2% in the POI group and 0.9 and 0.04% in the healthy group, respectively. Frequency of other candidate gene marker variants has been derived from studies involving around 100 subjects. The frequencies of variants range from 2 to 13% in the POI population and consistently stay around 0% in the control group (Table 2). Large-scale and multi-racial studies still need to be performed to further elucidate the prevalence of these genes in the POI and the control populations. Nevertheless, the current clinical studies provide support for consideration of use of genetic markers in the clinics.

**Fig. 2:**
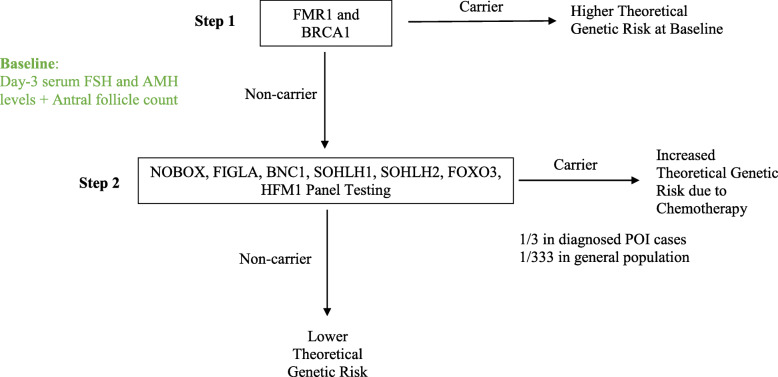
Proposed two-step screening algorithm to validate feasibility of genetic ovarian reserve testing in clinical practice. The flow chart demonstrates a cost-effective workflow to stratify patients into risk groups and facilitate evidence-based fertility preservation counseling. **Step 1**: FMR1 and BRCA1 mutation status are routinely tested in some clinics. FMR1 or BRCA 1 positive patients are at higher risk baseline. **Step 2**: Non-carriers of FMR 1 or BRCA 1 are triaged to undergo a 7-gene panel testing to further test for genetic variants to test their risk of loss of ovarian reserve after chemotherapy

**Fig. 3:**
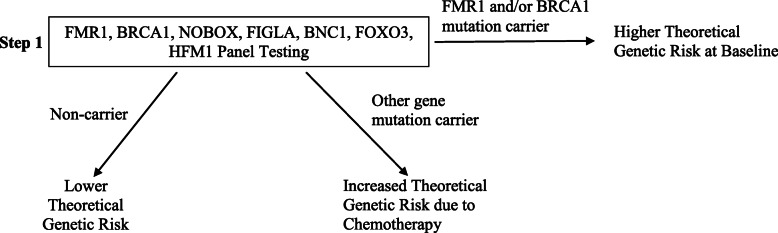
Proposed one-step gene panel ovarian reserve testing to assign patients to different risk groups. A 7-gene panel could be ordered routinely to determine the risk status for patients about to undergo chemotherapy and help accelerate evidence-based fertility preservation counseling without delaying treatments

Predictive genetic markers should have a high prevalence in the POI patients and a low prevalence in the healthy population. The biological basis for selecting the genetic markers needs to be supported by basic science. Based on these criteria, we propose a research screening algorithm to understand different levels of risk of loss of ovarian reserve among patients about to undergo chemotherapy (Fig. 2). The testing proposed will begin with screening of higher frequency pathogenic variants and progress to screening of lower frequency variants. The level of risk of each patient will be assessed based on panel results in addition to the other tests results such as day-3 serum FSH and AMH levels and antral follicle count. In the long run, a one-step 7-gene panel including FMR1 and BRCA1 with five other higher frequency variants may be developed and performed routinely to assess risk for every patient (Fig. 3).

### Applications of next generation sequencing (NGS) in ovarian reserve testing

**Fig. 4:**
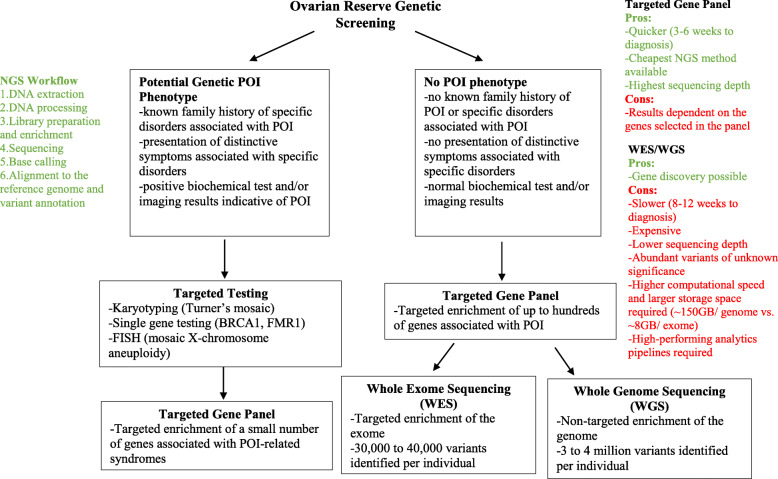
Theoretical NGS workflow to Pre-Chemotherapy Ovarian Reserve. Based on a patient’s medical background, different workflows employing NGS applications can be adopted to screen for genetic complement to predict future ovarian reserve of the patient

First-generation DNA sequencing using chain-terminator inhibitors has been widely used in diagnostic testing for the past 30 years since its invention in 1977 [101]. NGS has more recently begun to replace it in genetic testing due to its high speed and throughput [13, 102, 103]. Applications of NGS include targeted gene panel, whole exome sequencing (WES) and whole genome sequencing (WGS). These applications have contributed to the discovery of genes associated POI. Genetic etiology of POI may be monogenetic or polygenetic and can be broadly characterized in two categories: 1) genes and loci associated with POI and 2) genes and loci associated with disorders where ovarian insufficiency may be one of the symptoms [13, 15, 104]. Applications of NGS will not only continue to contribute to the discovery of genes associated with POI but also facilitate the ovarian reserve testing for patients with cancer prior to chemotherapy (Fig. 4). For example, targeted gene panel testing allows for a relatively quick and cost-effective way of screening of multiple genes associated with POI and provides more flexibility than single-gene testing. WES screens for protein-coding regions, approximately 1–2% of the genome and typically identifies 30,000 to 40,000 genetic variants that differ from the reference genome per person. WGS screens the entire genome including the non-coding regions and typically identifies 3–4 million variants per person [105, 106]. While a genome can now be sequenced within a day, the data sets generated by WES and WGS are high dimensional and complex in structure and requires continuous development of computing tools, platforms and guidelines around data security and infrastructure to reduce the cost of obtaining a complete disease profile from the raw data [107–110]. Despite the challenges, WES and WGS may be considered in cases where no diagnosis is obtained from targeted gene panel testing. NGS applications are the future of genetic diagnosis of patients who are susceptible to loss of ovarian reserve upon exposure to chemotherapy. We propose an initial adoption of targeted gene panel screening discussed below due to its lower cost and higher sequencing depth compared to WES and WGS. As the prices for WES and WGS decrease over time, they will be fully integrated into the clinical workflow and provide physicians with more diagnostic options.

### Screening

Obtaining a comprehensive picture of a patient’s genetic background enables more informative, evidence-based fertility preservation planning. In the long run, it is also likely to bring a positive economic impact on the cost of fertility preservation in female cancer patients. The average cost for a female cancer patient to preserve her fertility through oocyte or embryo cryopreservation was found to be $8655 by a study that surveyed 154 reproductive clinics in the US [111]. A recent study reported the cost of NGS infertility gene panel consisting of 87 genes to be $599 [112]. A 49-gene panel for genomic analysis of solid tumors was reported to cost around $449 [113]. The cost of gene panels consisting of less than 10 genes can be reasonably controlled to within $200 to $300 based on these results. In fact, multigene panel testing has recently been evaluated for its cost-effectiveness for all patients with breast cancer and one study in Norway found that a 7-gene panel with 5 non-BRCA genes was the optimal strategy with the highest incremental cost-effectiveness ratio [114, 115]. Given the current trend of moving towards precision reproductive medicine, commercial and institutional genetic panels for infertility risk assessment have started to emerge [116]. Performing multigene panel testing on patients indicated for chemotherapy can help clinicians stratify patients into risk groups and in the long run, provide targeted therapeutics for each patient. Due to the ethnic-group specific nature of current studies, we propose a trial 7-gene panel encompassing variants that appear at a high frequency specific to the ethnic group of the patient. For example, for a Han Chinese patient, a panel encompassing newborn ovary homeobox protein (NOBOX), folliculogenesis specific BHLH transcription factor (FIGLA), basonuclin 1 (BNC1), spermatogenesis and oogenesis specific basic helix-loop -helix 1 (SOHLH1), SOHLH2, FOXO3 and HFM1 may be ordered to identify patients susceptible to ovarian failure after chemotherapy that might not be apparent otherwise (Fig. 2). The prevalence of POI in the general population is 1% [117]. Assuming that having any one of the seven genetic variants is a mutually exclusive event, approximately 1 in 333 patients from the general population will be screened positive for one of the variants. Currently, the turnaround time for a multigene panel is between 4 and 6 weeks. For patients about to undergo chemotherapy, waiting for panel results could delay time-sensitive treatments. With advancement of technology, we expect the turnaround time to be shortened to between 3 and 4 days before multigene panel could be integrated into clinical onco-fertility practice. Furthermore, in the long run, a comprehensive panel testing encompassing all the genes known to be associated with POI should be examined (Tables 2, 3). Variants should be grouped according to their underlying mechanism of pathology. Carriers with more groups of variants may be at higher risk of developing ovarian failure compared to carriers with fewer groups of variants. Disease-inducing potential of each variant should be evaluated.

**Table 3:** Emerging Genes Associated with POI

Mechanism	Gene	Animal Study	Human Study
Cell-cycle progression; DNA damage response	NUP-107	Knockdown of NUP107 expression led to decreased expression of genes related to estrogen synthesis and receptors on granulosa cells which interferes their sensitivity to FSH [118]	A missense mutation of NUP107 was identified in two sisters with hypergonadotropic hypogonadism [119]
Regulation of ovulation rate; oocyte functional competence	BMPR2	A BMPR2 missense mutation led to aggregates localized at the endoplasmic reticulum in Chinese hamster ovary cell [120] BMPR2 is involved in signal transduction between oocytes and somatic cells [121, 122]	BMPR2 is implicated in folliculogenesis and human ovarian functions [120]
Meiosis	SYCE1	SYCE1 homozygous mutant mice failed to have offspring after 3 months and no follicles or oocytes were observed. Wildtype and heterozygous mutant females were normal [123]	A homozygous missense mutation was identified in two sisters with primary amenorrhea born to a consanguineous parents [63] SYCE1 mutation was found to be underlie an autosomal recessive pattern of POI [124]
	STAG3	STAG3 deficient female mice were found to be sterile with their fetal oocytes arrested at early prophase I. Their oocytes were found to be depleted at 1 week of age [125]	A truncating mutation was identified in two sisters with primary amenorrhea from a consanguineous Lebanese family [126] Two nonsense mutations were identified in two Caucasian sisters presented with POI [127] Two homozygous germline truncation mutations were identified in two sisters diagnosed with POI from a consanguineous Han Chinese family [128]
	MSH4	MSH4 knockout mice presented with meiotic failure and infertility. Many oogonia had been lost at 2-day postnatal detection. Ovaries were found to be small and contain few oocytes at 4 weeks [129]	A homozygous donor splice site mutation was found to cause POI [130]
	MSH5	MSH5 knockout mice was infertile and found to have a markedly reduced size of ovary with no developing follicles. At 2 months of age, no germ cells were found in these mice [131]	A homozygous missense mutation was identified in two Chinese sisters with POI [131]
	DMC1	DMC1 knockout mice presented with aborted oogenesis in embryos and no germ cells were found in adult mice ovary with a markedly reduced size of ovary. At 8-week postnatal evaluation, no follicles were found at any developmental stage [132]	A homozygous missense mutation was identified in a Chinese consanguineous family with POI phenotype [133]
	WDR62	WDR62 knockout mice exhibited meiotic initiation defects [134]	Two missense mutations were detected in two patients with POI [134]
Intercellular communication	GJA4	Connexin-37 is encoded by GJA4 gene. Connexin-37 deficient mice was found to lack mature follicles. They also failed to ovulate and developed numerous inappropriate corpora lutea [135]	A mutation was identified in 2 Caucasian patient with POI [136]
mRNA transcription; Cell growth and differentiation	POLR2C	POLR2C haploinsufficiency was found to disrupt rapid mRNA synthesis required during germ cell proliferation and oocyte maturation process in mice [137, 138]	A nonsense mutation was identified in a family with a dominant inheritance pattern of POI [139]
	POLR3H	Mice with the same missense mutation in POLR3H in patients with POI exhibited impaired reproductive function [140]	A homozygous missense mutation was identified in two unrelated families with idiopathic POI [140]
Germ cell development	MRPS22	Knockdown of MRPS22 in germ cells led to female sterility in drosophila [141]	Two homozygous missense mutations were identified in four females from two independent consanguineous families [141]
	NOTCH2	NOTCH2 knockout mice exhibited defective follicle development [142]	Two missense mutations were identified in patients diagnosed with POI [143]
Autophagy	ATG7/9	Germ-cell specific ATG7-knockout mice exhibited oocyte over-loss during neonatal period [144]	Two heterozygous missense mutations were identified in two patients diagnosed with POI [145]
Apoptosis; Cell cycle progression	TP63	TP63 protects female mice germline integrity during meiotic arrest [146]	
Homologous DNA repair	SPIDR	Meiotic RAD51 and DMC1 focus formation in response to DNA damage was found to be reduced in SPIDR knockout mice [147]	A homozygous nonsense mutation was identified in two daughters of consanguineous double first cousin parents of Arab ancestry, both diagnosed with POI [148]

#### Higher theoretical genetic risk at baseline

Certain genetic mutations such as FMR1 premutation and BRCA1 mutation have been shown to increase not only the risk of developing POI but also the risk of developing other pathologies. These genes should be screened routinely. For example, FMR1 premutation has been identified in 0.8 to 7.5% of cases of sporadic POI and up to 13% of cases of familial POI [149]. Prevalence of POI in carriers of FMR1 premutation has historically been shown to be between 13 to 26% [150, 151]. Another study, however, found it to be around 2% [98]. An accurate prevalence needs to be established through large-scale, population-specific studies. FMR1 premutation is also known to cause associated tremor/ataxia syndrome and a variety of phenotypes ranging from neuropathy to immune mediated disorder [152]. Similarly, BRCA1 mutation has recently been associated with POI but has long been screened at clinics as a marker of risk of developing breast cancer [153]. Carriers of FMR1 premutation or BRCA1 mutation are at high risk of developing ovarian failure regardless of chemotherapy. Prior to chemotherapy, carriers with age over 40 may have already presented with POI or other phenotypes. Prompt fertility preservation is encouraged for patients who wish to conceive in the future. Younger patients are likely to be asymptomatic and need to be identified through screening. Their risk of eventually developing ovarian failure and a potentially accelerated progression due to chemotherapy should be communicated. Fertility preservation in these patients should be discussed without delay.

#### Increased risk due to chemotherapy

Patients that screened negative for FMR1 and BRCA1 mutations requires further clinical investigation. Recent sequencing studies have identified several genetic variants such as NOBOX, FIGLA, SOHLH1, SOHLH2, FOXO3 and HFM1 that appear in high frequency in patients with POI. These genes have also been shown to be implicated in mechanism of ovarian failure. For example, NOBOX, FIGLA, SOHLH1, SOHLH2 and FOXO3 were found to be important in follicle development. HFM1 was found to be implicated in progression of meiosis. Screening of variants of the seven genes should be considered in patients who wish to conceive. Carriers of any one of the variants may be susceptible to exaggerated ovarian damage due to chemotherapy. Genetic profile of the variants complementary to medical history provides additional information to help patients gauge their risk of developing ovarian failure after chemotherapy and plan with clinicians about fertility preservation accordingly. In the future, a more comprehensive gene panel encompassing up to hundreds of genes may be tested to stratify patients in this group at a higher resolution. For example, carriers of both higher frequency and lower frequency variants may be at a different level of risk compared to carriers of higher frequency variants alone. As the penetrance and pathogenicity of each variant is better understood, such a comprehensive gene panel will provide detailed genetic profiles to guide fertility preservation practices.

#### Lower theoretical genetic risk

Patients without presentation or familial history of POI that screened negative for FMR1, BRCA1 and the seven genes mentioned above may have a lower theoretical probability of developing ovarian failure after chemotherapy, given our current understanding. Screening of rare genes associated with POI might be considered, if given sufficient clinical suspicion of increased risk.

## Future studies

The mechanism of ovarian damage due to chemotherapy is still not completely understood. Animal studies provided evidence that support germ and stromal cell apoptosis and/or accelerated follicle activation as possible mechanisms. There has yet to be conclusive results elucidating to what extent each mechanism gives rise to human clinical manifestation of ovarian failure. Future animal studies include co-immunohistological staining using markers of apoptosis and follicle activation on ovarian tissue after chemotherapy. Additional or alternative mechanisms might also arise through further investigation. Furthermore, human studies are essential to understanding the mechanism of damage. For example, biopsies of ovarian tissue in patients who experience ovarian failure after chemotherapy and need to undergo surgery may be collected and stained for markers of apoptosis and follicle activation. Staining results may further be correlated with genetic profile to validate the screening process proposed above. We expect patients with genetic variants implicated in DNA repair such as BRCA1 and MCM8/9 to present with strong markers for apoptosis and with those implicated in follicle activation such as FOXO3, BMP15 and SOHLH1 to present with strong markers for follicle activation. In addition, a patient’s genetic profile should be evaluated in conjunction with day-3 serum FSH and AMH levels as well as antral follicle count to understand if any correlations between genetic test results and these other test results exists.

Deep genetic sequencing should be performed on women with idiopathic POI to continue to uncover associated genetic variants at high resolution. As these genetic variants emerge, large scale, ethnic-group specific screening studies should be performed to investigate the frequency of these variants. Current studies are largely limited to about 100 patients and to certain ethnic groups (Table 2). These preliminary studies showed that some genetic variants are specific to ethnic groups. In addition, the frequency of individual variants in the control group in these studies are close to 0%. If future studies were to confirm these findings, a patient-centered variant screening program rather than a one-for-all routine screening proposed above would need to be established. Alternatively, specific patient groups may be selected with a recommendation of undergoing a genetic panel test.

## Conclusion

Certain chemotherapeutic agents have been documented to induce a high rate of ovarian failure in patients (Table 1). Yet, the effects of the ovarian reserve by the agents vary from patient to patient. This makes counseling and planning for fertility preservation challenging. Ovarian reserve testing using clinical markers predicts ovarian function after chemotherapy based on a patient’s baseline ovarian reserve. But some patients with high baseline ovarian reserve have poor outcomes after treatment. This discrepancy points to the need for a more predictive marker for a patient’s reproductive potential after chemotherapy. Genetic markers hold the promise to fulfil this need. As the mechanism of chemotherapy-induced ovarian damage continues to be investigated, genetic variants underlying these pathways may reliably predict reproductive potential based on basic mechanisms. Variants that appear in high frequency can be incorporated into routine screening in addition to molecular markers to help patients assess their risk.

## Data Availability

Not applicable.

## Abbreviations

KO: Knockout
WT: Wildtype
AMH: Anti-Mullerian hormone
PUMA: P53-upregulated modulator of apoptosis
PARP: Poly-ADP ribose polymerase
PI3K: Phosphatidylinositol 3-kinase
PTEN: Phosphatase and tensin homolog
FOXO3a: Forkhead box o3
TSC1,2: Tuberous sclerosis 1, 2
MCM8,9: Minichromosome maintenance 8, 9 homologous recombination repair factor
POI: Primary ovarian insufficiency
STAG3: Stromal antigen 3
HFM1: Helicase for meiosis 1
NUP107: Nucleoporin 107
SYCE1: Synaptonemal complex central element protein 1
NANOS3: Nanos C2H2-type zinc finger 3
PGRMC1: Progesterone receptor membrane component 1
BMP15: Bone morphogenic protein 15
FMR1: Fragile X mental retardation 1
FSH: Follicle-stimulating hormone
NGS: Next-generation sequencing
WES: Whole-exome sequencing
WGS: Whole-genome sequencing
NOBOX: Newborn ovary homeobox
FIGLA: Folliculogenesis specific BHLH transcription factor
BNC1: Basonuclin 1
SOHLH1,2: Spermatogenesis and oogenesis specific basic helix-loop-helix 1
